# Effect of UV-C Radiation on 3D Printed ABS-PC Polymers

**DOI:** 10.3390/polym15081966

**Published:** 2023-04-21

**Authors:** Catalin Gheorghe Amza, Aurelian Zapciu, Florin Baciu, Constantin Radu

**Affiliations:** Faculty of Industrial Engineering and Robotics, University Politehnica of Bucharest, 060042 Bucharest, Romania; aurelianzapciu@yahoo.com (A.Z.); florin.baciu@upb.ro (F.B.); rcdnd@yahoo.com (C.R.)

**Keywords:** 3D-printing polymers, ABS-PC, PCABS, accelerated aging, ultraviolet, UV-C

## Abstract

During the initial stages of the COVID-19 pandemic, healthcare facilities experienced severe shortages of personal protective equipment (PPE) and other medical supplies. Employing 3D printing to rapidly fabricate functional parts and equipment was one of the emergency solutions used to tackle these shortages. Using ultraviolet light in the UV-C band (wavelengths of 200 nm to 280 nm) might prove useful in sterilizing 3D printed parts, enabling their reusability. Most polymers, however, degrade under UV-C radiation, so it becomes necessary to determine what 3D printing materials can withstand the conditions found during medical equipment sterilization with UV-C. This paper analyzes the effect of accelerated aging through prolonged exposure to UV-C on the mechanical properties of parts 3D printed from a polycarbonate and acrylonitrile butadiene styrene polymer (ABS-PC). Samples 3D printed using a material extrusion process (MEX) went through a 24-h UV-C exposure aging cycle and then were tested versus a control group for changes in tensile strength, compressive strength and some selected material creep characteristics. Testing showed minimal mechanical property degradation following the irradiation procedure, with tensile strength being statistically the same for irradiated parts as those in the control group. Irradiated parts showed small losses in stiffness (5.2%) and compressive strength (6.5%). Scanning electron microscopy (SEM) was employed in order to assess if any changes occurred in the material structure.

## 1. Introduction

In the early weeks of the COVID-19 pandemic, healthcare facilities were overwhelmed by the influx of people requiring medical assistance, leading to problems with personal protective equipment supply and availability, as stated by the World Health Organization [1]. These shortages were widespread and happened at all levels of medical care, from nursing homes, local hospitals and clinics to emergency rooms at some of the world’s biggest hospitals [2,3,4]. The distribution of locations that needed equipment and the diversity of items needed made resupplying extremely difficult, both from a manufacturing perspective and from a logistical one [5,6,7,8]. The necessary equipment had to be manufactured quickly and had to be distributed widely, which led to even more problems occurring because of resource misallocation [9,10]. Additive manufacturing (AM), also known as 3D printing, has a decentralized aspect and a unique ability to adapt to producing new part designs without additional capital investment [11,12,13,14]. With AM, parts can be produced on-site and on-demand, lowering the delay between necessity and availability. For this reason, 3D printing saw increased use during the initial responses to the medical equipment shortages [15,16,17]. Hospital technicians, small businesses and even private citizens who owned hobby-grade 3D printers began creating, distributing and manufacturing designs of protective equipment [18,19,20].

While the majority of the manufactured products meant to replace medical equipment in case of critical and urgent supply issues were designed for single use (e.g., swabs for nasopharyngeal testing [21,22,23]), other products could be used multiple times if sterilized under inadequate conditions. Examples of such products are face shields [24,25].

Material Extrusion 3D Printing (MEX) is an additive manufacturing process [26] that uses thermoplastic material feedstock. MEX 3D printing enables several advantages over more traditional manufacturing.

Studies performed on N95 respirators during the COVID-19 pandemic showed that filtering efficiency and infection prevention increase when the respirator is properly fitted [27,28,29]. The fact that 3D printing can be used to rapidly manufacture bespoke products for each healthcare worker can be leveraged to provide better and tighter seals for respirators. Ballard et al. produced respirators using real 3D data obtained using computer tomography that successfully passed OSHA-certified testing [30], while McAvoy et al. produced polymer frames that improve the fit of available N95 respirators [31].

In the medical field, the sterilization of medical equipment is performed through various methods, such as autoclaving, dry heating, ultrasonic sterilization [32,33,34,35], or chemical sterilization using various chemicals, such as hydrogen peroxide or ethylene oxide [36]. However, some of these methods are not applicable to certain polymers. For example, acrylonitrile butadiene styrene (ABS) parts deform when going through repeated autoclaving treatments [37] and suffer from a degradation of mechanical properties when sterilized using alcohol or other disinfectants [38,39].

In recent years, researchers have paid increasing attention to the sterilizing action of ultraviolet radiation, specifically UV-C radiation [40,41,42,43]. The International Commission on Illumination (in French: Commission Internationale de l’Eclairage, CIE) uses a wavelength of 280 nm to separate UV-C and UV-B bands, 315 nm to separate UV-B and UV-A bands, and 400 nm to separate UV-A and photosynthetically active radiation. The effects of UV-B/UV-A radiation have been studied extensively, as radiation of these wavelengths reaches Earth’s troposphere naturally. However, UV-C radiation is entirely filtered by the Earth’s atmosphere and has to be produced artificially. For this reason, there is no accelerated aging testing standard for the action of UV-C on materials, unlike that for radiation of longer wavelengths [44]. A workshop organized by the United States National Institute of Standards and Technology (NIST) and the International UV Association (IUVA) [45] aimed to gather information about applications, new certifications and new guidelines regarding UV-C disinfection and sterilization [46]. This workshop also highlighted the need to study the effects of UV-C radiation on materials. Standards for UV-C applications and testing are currently under development as a cooperation between NIST and the Illuminating Engineering Society (IES.org).

To get ahead of this trend, materials testing is important in determining their applicability and compatibility with novel sterilization methods. In the case of UV-C sterilization, materials testing is also crucial to the standardization efforts.

Acrylonitrile Butadiene Styrene (ABS) is a 3D printing material with widespread use [47] due to its good mechanical characteristics [48,49] and low cost. The material is known for its weak ultraviolet resistance [50]. Through the addition of polycarbonate fibers (PC) to an ABS base, manufacturers have successfully created a copolymer blend with enhanced mechanical properties and ultraviolet resistance [51] while still maintaining its ease of processing through MEX 3D printing [52]. This copolymer has high strength and stiffness, high heat resistance [53,54], and good impact resistance [55]. The newly created acrylonitrile butadiene styrene and polycarbonate polymer blends (ABS-PC) are being marketed for use in industrial applications, prototyping, toolmaking or end-use parts manufacturing [56].

Given this context, this work investigates the changes in the mechanical behavior of 3D-printed parts made from ABS-PC after exposure to artificial UV-C radiation. This work pursues one of the research directions raised by the NIST-IUVA workshop highlighted previously related to identifying and studying suitable materials. Unlike more common polymers, such as ABS, polylactic acid (PLA), or modified polyethylene terephthalate-glycol (PETG), literature on 3D printed ABS-PC blends is scarce and does not include mechanical behavior after exposure to artificial radiation in the UV-C spectrum. Additionally, the study of 3D printed materials is necessary for the implementation and approval of 3D printing techniques and 3D printed devices for medical applications, including those produced at the point of care (3DPOC) [57,58]. By using a controlled irradiation treatment in an irradiation chamber, an accelerated effect that simulates many cycles of ultraviolet sterilization can be observed. The mechanical properties assessed in this work are tensile and compression strength, material stiffness, as well as changes in creep behavior of ABS-PC samples subjected to prolonged tensile loads.

## 2. Materials and Methods

Accelerated aging of materials under UV-C radiation does not have well-defined testing standards. Testing parameters and procedures should be determined based on existing literature. An appendix of the ISO 4892-2 standard [59] mentions modifying testing conditions to use a mercury lamp that generates 10 W/m^2^ of 254 nm wavelength radiation, a wavelength that falls in the UV-C band. A testing protocol for the assessment of material behavior under UV-C radiation is defined by the Business and Institutional Furniture Manufacturers Association (BIFMA, Grand Rapids, MI, USA) [60]. These guidelines were created for healthcare furniture design and specify that materials should be tested with 291 kJ/m^2^ of UV-C radiation for a period of 12 to 24 h.

The material analyzed in this study is a copolymer made from ABS (Acrylonitrile Butadiene Styrene) and PC (PolyCarbonate). The tested material is commercially available under the brand name Z-PCABS, manufactured by Zortrax (Olsztyn, Poland). According to the manufacturer, this Z-PCABS blend contains 55–60% ABS, 30–35% PC and up to 10% additives and colorants. The material has a glass transition temperature of 104 °C and melts at a temperature of 260 °C (manufacturer specifications). The rated tensile strength is 36.89 MPa (ISO 527:1998 [61]), and the material’s specific density is 1.14 g/cm^3^. The material was sourced as a 1 kg spool of 1.75 mm diameter filament, opaque, ivory color. The spool of material was provided in a sealed reflective bag that included silica desiccant and was unsealed only prior to printing. Decisive in material selection was the fact that many filament manufacturers have settled on this composition based on process-specific requirements. When used for 3D printing, ABS should be extruded at temperatures between 230 °C and 260 °C while PC requires a higher temperature, 260 °C to 300 °C. As PC content in the blend increases, so does the temperature needed to process the material. The selected ABS/PC ratio places this material at the upper end of what common 3D printers can process in terms of extrusion temperature. Above this temperature, MEX 3D printers need to be equipped with high-temperature resistant components, higher-power ceramic heaters, temperature sensors for high-temperature applications and heated enclosures.

All specimens were 3D printed on a Zortrax M200 3D printer equipped with a 0.4 mm diameter nozzle. Z-Suite software, produced by the same company, was used to section the virtual model into layers. Printing parameters were selected based on what is known to provide consistent printing results [62,63,64,65] in order to minimize potential errors related to the manufacturing process. Layer settings used for slicing are layer height = 0.19 mm and layer width = 0.4 mm, with 2 outside perimeters and solid infill using a grid pattern (45° raster angle, alternating each layer [66]). The extrusion temperature was set at 265 °C, and the build platform temperature was set at 85 °C. Parts were printed horizontally [67]. Table 1 shows some of the process parameters used for 3D printing test samples.

**Figure 1:**
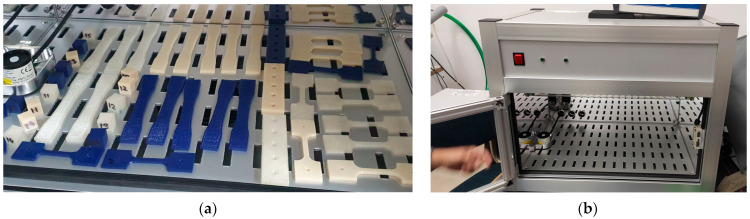
Accelerated aging in irradiation chamber (**a**) Test parts positioned for irradiation cycle; (**b**) Irradiation chamber.

Considering the aspects mentioned above, the 3D-printed test samples (Figure 1a) were subjected to ultraviolet radiation in an Opsytec Dr. Grobel BS-02 irradiation chamber (Ettlingen, Germany) (Figure 1b). The irradiation chamber comes equipped with two groups of fluorescent lamps, one group emitting UV-C, λ = 254 nm, and the second group emitting UV-B λ = 315 nm. The radiation dose is measured with calibrated sensors and controlled using a UV-MAT controller produced by the same manufacturer [68].

A group of 3D-printed samples was exposed to 254 nm radiation for 24 h. During the irradiation cycle, a back panel temperature of 50 °C was maintained in the chamber, and the samples were hit with 10 W/m^2^ radiating power. After 24 h, the lamps were turned off, and the test samples were left in the chamber for an additional 4 h to cool down to room temperature. In order to have another reference point for determining radiation effects on the material, a second set of sample parts were exposed to UV-B radiation in the same irradiation chamber.

The effects of UV-B and UV-C radiation were analyzed according to ISO 4892-1:2016, which indicates how data from accelerated aging using light radiation exposure [69] should be analyzed. Tensile and compressive strength tests were designed according to ISO 4582:2017 [70].

For tensile strength determination, 15 specimens were 3D printed from Z-PCABS according to ASTM type I dimensions [71].

For compressive strength tests, 15 test samples measuring 15 mm × 15 mm × 15 mm were 3D printed from the same material.

For each mechanical property test, the samples were split into 3 groups, forming groups of 5 randomly selected samples.

For strength testing (tensile strength, compressive strength), accelerated aging using UV-B radiation using the parameters highlighted previously was performed on the first group of samples. The second group was exposed to UV-C radiation. The third group did not undergo any radiation exposure and represented the control group. Before and after the accelerated aging treatment, all samples were measured using electronic calipers and were visually inspected.

**Figure 2:**
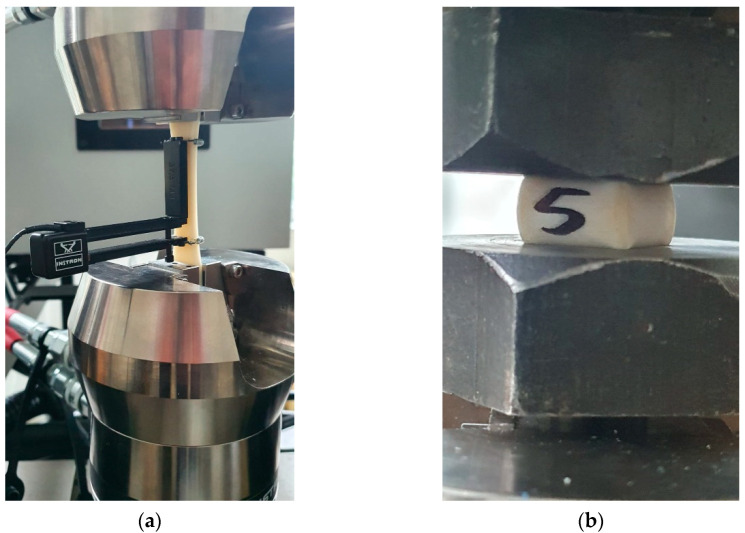
Mechanical strength tests: (**a**) Tensile strength testing of 3D printed dog-bone ABS-PC samples; (**b**) Compression strength testing of 3D printed cubic ABS-PC samples.

In total, 15 3D-printed samples (dog bone-shaped) were tested for tensile strength on an Instron 8872 machine (Norwood, MA, USA) (Figure 2a). Tests were performed starting with a preload force of 5 N with 1 mm/minute loading speed. Elongation of the test part under tensile load was measured using an electronic extensometer and was used to determine part stiffness. Fifteen 3D-printed cubic samples were tested for compressive strength on an Instron 8801 machine (Norwood, MA, USA) (Figure 2b). The compressive force is applied along the sample Z-axis, perpendicular to the horizontal 3D-printed layers. Preload for this test was 5 N. All strength tests were in controlled conditions of 50% RH at 24 °C.

Section 3.1 details the results of tensile tests (strength, stiffness), while Section 3.2 discusses the results of compressive tests (strength).

**Figure 3:**
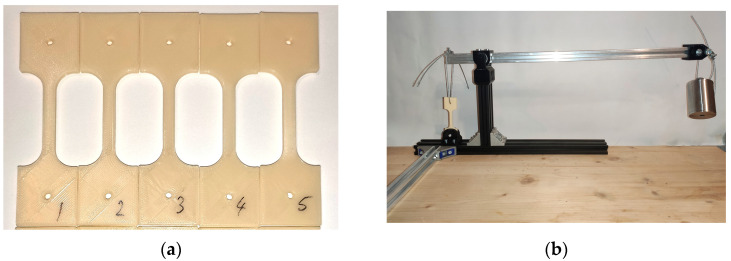
Creep properties testing: (**a**) 3D printed parts made for tension creep testing; (**b**) Testing rig for determining tensile creep.

Polymers and fibers have a known tendency to deform plastically when subjected to loads for long periods of time, a characteristic commonly referred to as cold flow or creep. For certain polymers, creep-inducing stress can occur even at a small fraction of the material’s ultimate strength. ABS-PC blends experience creep, with creep resistance increasing with increased PC content [72,73]. Thus, the effects of radiation on the creep behavior of ABS-PC parts was also investigated. Tensile creep testing was performed on 10 3D-printed specimens with a narrow section of 5.2 mm × 3 mm (Figure 3a). Testing was performed in compliance with standard ASTM D2990-17 [74]. The creep behavior of parts exposed to UV-C radiation was compared to that of parts in a control group.

For finding creep under tensile load, parts were loaded in tension in a purposefully designed rig (Figure 3b). Creep was determined by applying a load that produces mechanical stress of 25% of the ultimate tensile strength measured previously for parts in the control group. Sample elongation under this tensile load was measured after 2 h, 6 h, 21 h, 24 h, then once every 24 h for a total of 168 h (7 days). The distance between the ends of the samples was measured using a micrometer on each side of the narrow section (left and right sides). The average change in these two distances was considered as elongation. The results of creep testing are discussed in Section 3.3.

The exterior surfaces and internal structure of the samples were analyzed using Scanning Electron Microscopy analysis (SEM). One sample each was selected from the group exposed to 254 nm radiation and from the control group. The analysis was performed using a Quanta Inspect F50 scanning electron microscope from Thermo Fisher Scientific (Eindhoven, The Netherlands) at a resolution of 1.2 nm. A layer of Au was sputter-coated on the samples (coverage time 90 s) using a Quorum Technologies Q150 PlusSeries coater (Lewes, UK). Section 3.4 discusses SEM results.

## 3. Results

Dimensional and visual checks were performed on the parts before and after accelerated aging under 254 nm radiation. Dimensions of the UV-C exposed parts were measured using electronic calipers before and after irradiation, revealing no statistically relevant changes in dimensions after UV-C exposure. Table 2 shows the average dimensions of samples in the aged group vs. the control group.

### 3.1. Tensile Strength and Young’s Modulus

**Figure 4:**
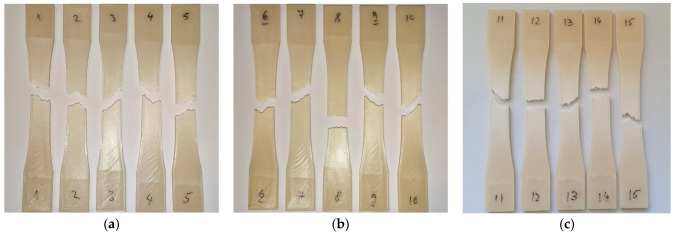
Fracture modes of tensile-tested parts: (**a**) Fractured ABS-PC parts from the control group; (**b**) Fractured ABS-PC parts exposed to UV-B; (**c**) Fractured ABS-PC parts exposed to UV-C.

Visual analysis of the failure mechanism found that the ABS-PC samples fractured along the deposited polymer filaments, creating a zig-zag failure pattern. The same pattern was observed in the control group and both groups exposed to radiation (UV-B/UV-C) (Figure 4). This fracture pattern can be explained by considering the anisotropic mechanical properties of parts produced through MEX 3D printing [75]. The anisotropy is caused by higher tensile strength along the filaments than the tensile strength of the adhesion between adjacent filaments [76] and is influenced by print orientation, infill type and amount, raster angle, and layer height [77,78,79]. The similar failure modes for all tested groups also indicate that changes generated by the irradiation treatments were uniform throughout the parts. The parts displayed a slight browning of the ivory-colored material following irradiation. A similar amount of browning is found in both UV-B and UV-C groups.

**Figure 5:**
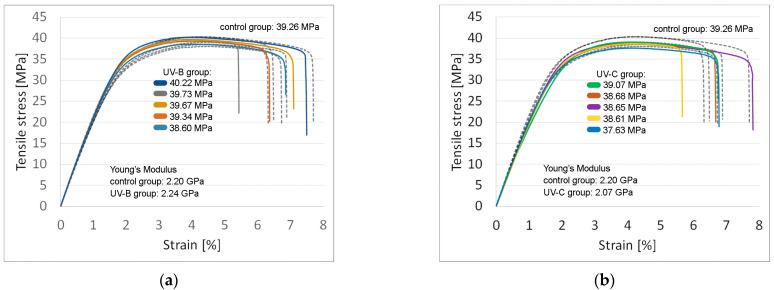
Stress-strain diagrams (tensile tests): (**a**) Samples exposed to UV-B λ = 315 nm (solid line) and samples from the control group (dashed line); (**b**) Samples exposed to UV-C λ = 254 nm (solid line) and samples from the control group (dashed line).

Experimental data from tensile testing of PC-ABS are shown in Figure 5 as stress-strain graphs.

Dog bone-shaped samples made from ABS-PC subjected to the previously described dose of UV-B radiation had no statistically significant difference in tensile strength (39.51 MPa vs. 39.26 MPa; *F* = 0.23, *p* = 0.65) and stiffness (2238.06 MPa vs. 2196 MPa; *F* = 1.57, *p* = 0.24) compared to the parts in the control group.

Parts made from ABS-PC aged under UV-C exhibited 1.86% lower tensile strength compared to control samples (38.53 MPa vs. 39.26 MPa), a result that is not statistically significant (*F* = 1.91, *p* = 0.20). On average, the Young’s Modulus of aged samples decreased by 5.51% vs. control samples (2075 MPa vs. 2196 MPa), a result that was determined to be statistically significant (*F* = 5.70, *p* = 0.04).

Results for average tensile strength after calculating the standard error can be found in Table 3. The same table shows the average Young’s Modulus.

### 3.2. Compressive Strength

**Figure 6:**
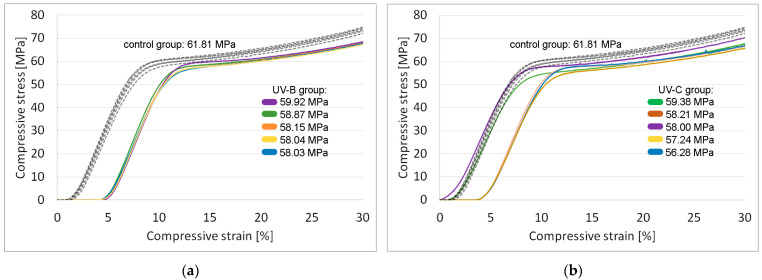
Stress-strain diagrams (compressive tests): (**a**) ABS-PC samples exposed to UV-B λ = 315 nm (solid line) and control samples (dashed line); (**b**) ABS-PC samples exposed to UV-C λ = 254 nm (solid line) and control samples (dashed line).

Compressive loads applied during compressive strength testing deformed the parts plastically without visible material rupture at their surface. Compared to parts in the control group (no UV exposure), all samples in the accelerated aging groups performed worse. Overall, ABS-PC samples have 5.2% lower compressive strength after UV-B irradiation (58.60 MPa vs. 61.81 MPa; *F* = 33.8, *p* = 4 × 10^−4^) while samples subjected to UV-C radiation have 6.5% lower compressive strength than those in the control group (57.82 MPa vs. 61.81 MPa; *F* = 36.3, *p* = 3 × 10^−4^). Stress-strain diagrams for ABS-PC are shown in Figure 6.

Results for average compressive strength after calculating standard error are shown in Table 4.

### 3.3. Creep Characteristics of the Analyzed Material

Tensile creep testing was performed with loads appropriate to the tested material based on the tensile strength value identified previously (σ_Z-PCABS_ = 39.26 MPa). The parts are placed in a testing rig that uses mechanical advantage to amplify the load applied to one of its ends. The cross-section area of the tested parts is 15.6 mm^2^ (5.2 mm × 3 mm). Table 5 shows the main parameters (loads) used in this experiment.

**Figure 7:**
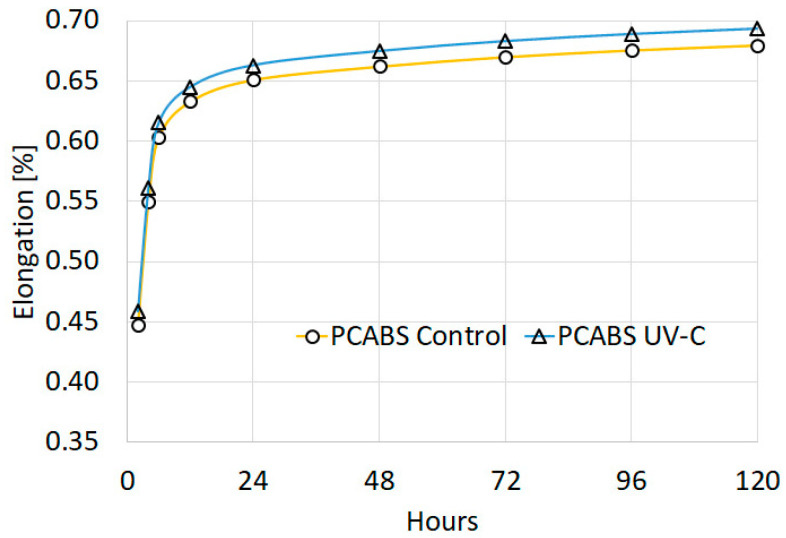
Tensile creep of Z-PCABS; graph showing an increase in elongation over time.

Figure 7 shows the tensile creep of ABS-PC samples while being loaded (9.82 MPa) over a period of 168 h. The majority of the elongation took place right after the load was applied, and the rate of creep continued to slow down with the rise in strain. Despite the fact that the parts from the UV-C exposed group experienced more deformation under prolonged tensile stress, they exhibited very similar creep curves.

### 3.4. Scanning Electron Microscopy (SEM)

**Figure 8:**
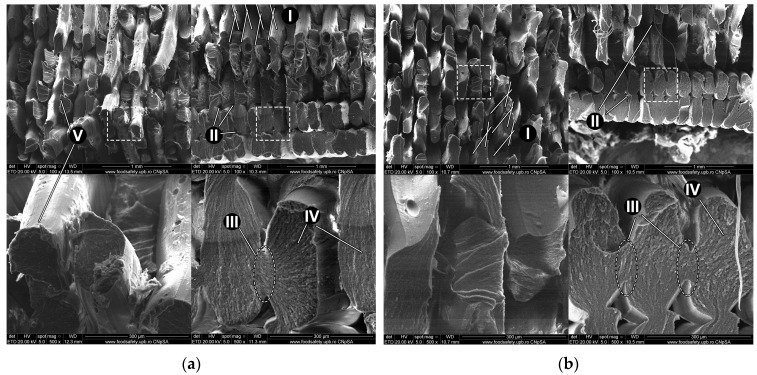
SEM imaging of fractured ABS-PC sample with the left column showing rupture surfaces in part infill; right column showing rupture surface in part contour: (**a**) Z-PCABS sample from the control group; (**b**) Z-PCABS sample exposed to UV-C λ = 254 nm; I—fracture parallel with filaments; II—microvoids; III—interlayer fusion; IV—extensive flaking at the fractured surface; V—reduced necking of the ruptured filaments.

To gain insight into how the samples failed under tensile stress and to spot any potential changes in the internal structure following exposure to UV-C radiation, a fractographic analysis was performed using SEM. In Figure 8a, an SEM image is presented, depicting an ABS-PC sample from the control group. Figure 8b displays an SEM image of a sample from the irradiated group (UV-C).

The ABS-PC samples fractured along the deposited filament lines (Figure 8a,b-I), as previously described in Section 3.1. Overlap of the deposited filaments, along with flattening following deposition, can be observed, giving the filaments a characteristic oblong shape. The MEX process introduces in the manufactured parts specific gaps usually referred to as “microvoids”. These voids are triangular in shape due to the angled raster used with infill deposition and the oblong shape of deposited filaments and form weak points in the material structure [80]. The formation of microvoids is generally associated with the lack of pressure applied to the molten filament while it is being deposited, unlike other manufacturing processes, such as injection molding [81]. Microvoids can be observed in both SEM-analyzed samples, with or without radiation exposure (Figure 8a,b-II).

Interlayer adhesion is distinctly noticeable in both the control sample (Figure 8a-III) and the radiation-aged sample (Figure 8b-III). Samples from both groups present flaking at the rupture surface with small areas of smooth fracture surface (Figure 8a,b-IV), with the control part also showing reduced necking of ruptured filaments (Figure 8a-V). This indicates the parts do not display increased brittleness after 24 h of UV-C exposure.

## 4. Discussion

This work investigated how sterilizing UV-C radiation λ = 254 nm affects the mechanical properties of ABS-PC components after 24 h of exposure. This is particularly significant when considering the growing use of 3D printing for functional parts. The study evaluated the tensile and compressive strength of samples that were 3D printed using the same process parameters and then exposed to 2 different radiation wavelengths (UV-B λ = 315 nm, UV-C λ = 254 nm). The group of parts exposed to a UV-C accelerated aging cycle was compared to a control group.

Following exposure to UV-C radiation ABS-PC parts produced a change in color at their surface, consistent with the degradation of the ABS component [82]. The changes were only visual, with no dimensional differences between parts from both groups. This is true for both bulky parts like the compressive strength tested specimens (15 mm × 15 mm × 15 mm cubes) and for high length-to-width ratio parts like the samples used for tensile strength testing (115 mm × 13 mm dog bone shape).

Laureto et al. [83] found that a difference exists between ASTM D638-14 Type I and Type IV dimensions when testing 3D printed parts, with the type I test specimens producing slightly better results tensile strength results. However, the difference in specimen performance is not expected to influence the conclusions drawn in this paper.

Following tensile strength testing, there was no statistically significant difference between unexposed samples and samples exposed to 24 h of 10 W/m^2^. UV-C radiation. A decrease in stiffness was observed after irradiation which can be attributed to the scission of molecular bonds.

Compressive strength testing showed a weakening of ABS-PC following radiation exposure, with aged parts (UV-C) having 6.5% less compressive strength.

Tensile creep testing resulted in similar elongation-time curves for the control group and the irradiated group, mirroring the findings regarding material stiffness. Mohamed et al. found that different printing parameters influence the creep resistance of PC-ABS 3D printed parts [84], a fact that highlights the need for further testing with other parameter sets. The amount of creep and the creep rate should only be associated with the particular use of a 45°/−45° infill raster angle. As Zhang et al. have found, the creep resistance of MEX 3D-printed parts is anisotropic and is influenced by infill orientation, with parts printed using 90° infill having the highest creep resistance [85]. However, infill orientation is not expected to produce different relative results (unexposed parts vs UV-C exposed).

SEM imaging was used to investigate whether any changes in the material occurred. Samples from both groups presented features common to the manufacturing process, such as microvoids and interlayer fusion regions. Key aspects commonly used to determine material behavior, such as fracture appearance and fracture surface smoothness, were also similar, mirroring the small differences observed following destructive testing.

UV radiation can cause polymers to age by breaking down their molecular chains, weakening their physical properties. This is because UV radiation contains high-energy photons that are capable of breaking chemical bonds in the polymer chains. When this happens, the polymer molecules become shorter, and the polymer chains become weaker, which can lead to the degradation of the material over time. This process is often referred to as photo-oxidation. One of the primary ways photo-oxidation degrades the material is by initiating the formation of free radicals—highly reactive molecules that can cause chain reactions in the polymer—leading to the formation of new chemical bonds and altering the polymer’s structure. In the presence of oxygen, free radicals can also react with oxygen molecules to form peroxides. Peroxides can then react with other polymer molecules to form more free radicals, perpetuating the chain reaction and causing further degradation of the polymer. With short wavelength radiation, such as UV-C, another important effect occurs, namely Fries rearrangement. When high-energy photons are absorbed by the polymer, primarily free radicals can form by breaking carbonate bonds in a process that does not need the presence of oxygen [86].

It is known that PC has better UV resistance compared to non-oxygen polymers, such as ABS [87,88]. We can hypothesize that the good behavior of ABS-PC is not only due to the PC component being more UV resistant, but its presence could aid the ABS component as well. It is known that at low wavelengths, ultraviolet radiation consists of photons with sufficient energy to break molecular bonds, creating cross-linking and scission effects on polymers [89,90]. It is, thus, possible for the radicals formed in the PC component to have a scavenging effect on other free radicals formed during the cleavage of molecules in the ABS component. This hypothesis requires further investigation.

In addition to causing physical changes in the polymer, UV radiation can also affect its optical properties. Polymers that are exposed to UV radiation can become discolored or yellowed (as was observed in these experiments), as photons can break down chromophores, which are chemical groups responsible for a polymer’s color. This is an important aspect to consider when designing parts where functionality depends on the material’s optical properties.

A large compilation of 431 studies on the UV dose (fluence) needed to inactivate common pathogens reveals that most pathogens see a 1-log reduction in numbers (90% pathogen inactivation) at UV doses lower than 20 mJ/cm^2^ [91,92]. This is equivalent to 20 s of exposure to the UV-C radiation source used in this experiment. These factors indicate that the investigated aging treatment is relevant to the common practices of sterilizing materials and surfaces. Given the good response of ABS-PC 3D-printed parts in terms of tensile and compressive strength, stiffness and creep behavior before and after UV exposure, this material should be considered for applications where UV sterilizing is employed. This study looked at the performance of a common commercially available ABS-PC blend. As discussed previously, a higher ratio of PC would increase UV stability while decreasing processability through MEX 3D printing. Further improvement of the material’s performance and stability under ultraviolet radiation could be obtained through various methods. Additives, such as organo-modified layered double hydroxides are known to increase the photostability of polymers while providing anti-bacterial properties [93]. Other UV absorbers and UV stabilizers can also be used [94]. Another aspect worth studying is the replacement of ABS with acrylonitrile styrene acrylate (ASA) in a PC/ASA copolymer. ASA has superior UV resistance compared to ABS, but a PC/ASA copolymer requires higher processing temperatures (275 °C extrusion temperature, 110 °C bed temperature). It is important to note that the presence of MEX process-specific features, such as layer lines, microvoids, etc., may play a role in determining the sterilization efficiency of 3D-printed parts, and future studies need to address this fact.

## 5. Conclusions

The objective of this paper was to examine the impact of UV-C exposure-induced accelerated aging on the mechanical characteristics of ABS-PC copolymer samples manufactured through MEX 3D printing. Mechanical testing found minor tensile and compressive strength decreases following irradiation. The same tests found that UV-C radiation has slightly decreased part stiffness.

After conducting a tensile creep test, it was observed that the creep behavior remained unchanged even after exposure to UV-C radiation, and both exposed and unexposed materials exhibited comparable creep curves.

The results overall suggest a minor reduction in mechanical properties of 3D printed ABS-PC parts following an irradiation treatment. These minor changes in mechanical properties, coupled with the proven long-term stability of ABS-PC blends, suggest that these materials are suitable for use in the presented scenarios. This is also true for parts subjected to continuous tensile stress and vulnerable to polymer creep. SEM investigation mirrored the findings of destructive testing,

Testing is needed to assess the medical efficiency of ultraviolet UV-C sterilization on such parts, given the inherent characteristics of MEX 3D printed materials. Elements, such as surface porosity, hygroscopy, etc., may represent a hurdle in the adoption of these materials.

It is important to mention that variations in feedstock material compositions, 3D printing equipment and software, and different printing parameters may yield slightly different outcomes for MEX 3D printed parts, as emphasized by Popescu et al. in a review of mechanical property testing [95]. In-house assessment of material properties, 3D-printing machine characteristics and parameter sets is recommended before producing end-use polymer parts.

## Figures and Tables

**Table 1:** Main parameters used in the 3D-printing process for sample manufacturing.

Material	Layer Height	Perimeters	Infill	Infill Pattern	Extrusion Temperature	Bed Temperature
Z-PCABS	0.19 mm	2	100%	“Grid” 45°/−45°	265 °C	85 °C

**Table 2:** Sample dimensions were measured using electronic calipers.

	Tensile Strength Sample		Compression Strength Sample	
	Width	Height	X	Z
3D model [mm]	13.00	4.20	15.00	15.00
Control samples [mm]	13.07 ± 0.012	4.15 ± 0.016	15.00 ± 0.016	15.16 ± 0.010
UV-C samples [mm]	13.09 ± 0.010	4.14 ± 0.010	15.01 ± 0.010	15.18 ± 0.012

**Table 3:** Experimental results—average tensile strength and Young’s Modulus.

Property	ABS-PC (no UV)	ABS-PC (UV-B)	ABS-PC (UV-C)
Tensile strength [MPa]	39.26 ± 0.47	39.51 ± 0.27	38.53 ± 0.24
Young’s Modulus [MPa]	2196.4 ± 31.7	2238.1 ± 9.78	2074.9 ± 39.8

**Table 4:** Experimental results—average compressive strength.

Property	ABS-PC (no UV)	ABS-PC (UV-B)	ABS-PC (UV-C)
Compressive str. [MPa]	61.81 ± 0.41	58.60 ± 0.37	57.82 ± 0.52

**Table 5:** Creep testing parameters.

Material	Test Parameter			
	Strength [MPa]	Creep Test Stress [MPa]	Targeted Tensile Force on Sample [N]	Test Rig Load Configuration
ABS-PC	39.26	9.82	154	22 N × 7

## Data Availability

Data will be made available on request.
